# Neural networks within multi-core optic fibers

**DOI:** 10.1038/srep29080

**Published:** 2016-07-07

**Authors:** Eyal Cohen, Dror Malka, Amir Shemer, Asaf Shahmoon, Zeev Zalevsky, Michael London

**Affiliations:** 1 Life Science Institute, Hebrew University, Jerusalem, Israel; 2 Faculty of Engineering, Bar Ilan University, Ramat Gan, Israel; 3 Faculty of Engineering, Holon Institute of Technology, Holon, Israel; 4 The Edmond and Lily Safra Center for Brain Sciences, Hebrew University, Jerusalem, Israel

## Abstract

Hardware implementation of artificial neural networks facilitates real-time parallel processing of massive data sets. Optical neural networks offer low-volume 3D connectivity together with large bandwidth and minimal heat production in contrast to electronic implementation. Here, we present a conceptual design for in-fiber optical neural networks. Neurons and synapses are realized as individual silica cores in a multi-core fiber. Optical signals are transferred transversely between cores by means of optical coupling. Pump driven amplification in erbium-doped cores mimics synaptic interactions. We simulated three-layered feed-forward neural networks and explored their capabilities. Simulations suggest that networks can differentiate between given inputs depending on specific configurations of amplification; this implies classification and learning capabilities. Finally, we tested experimentally our basic neuronal elements using fibers, couplers, and amplifiers, and demonstrated that this configuration implements a neuron-like function. Therefore, devices similar to our proposed multi-core fiber could potentially serve as building blocks for future large-scale small-volume optical artificial neural networks.

Networks consisting of thousands of densely interconnected neurons in our brain constitute the functional units that underlie cognitive processes^1^,^2^. These complex networks inspire brain researchers and engineers to develop mathematical models called artificial neural networks^3^,^4^. While these models are oversimplified compared to real neuronal systems, they still capture central functional properties of their biological counterparts^5^,^6^. Although, the initial popularity of this field^7^,^8^,^9^,^10^ declined we have only recently seen a resurgence of interest, following the design of deep networks, convolutional networks, and chaotic networks^11^,^12^,^13^,^14^,^15^,^16^,^17^. Recently there have been major efforts to transfer these designs into hardware implementation. The electronics industry have been developing neural-network-based hardware for the acceleration of specific tasks^18^. In addition, the field of neuromorphics, the development of brain inspired hardware systems, has been boosted via funding of mega-projects, pushing toward better and more realistic models^19^,^20^,^21^.

Optical neural networks had already been developed in the early 80’s^22^. These systems were capable of learning Boolean functions, face recognition, and handwriting deciphering^23^,^24^,^25^,^26^. These networks utilize the advantages of any optical device over electronic ones: the ability to transmit information from a single point source to any arbitrary number of targets, a task that requires large wire-volume in electronics. Additionally, optical systems generate less heat than electronic ones. A common drawback of all these optical implementations, however, is their large size and lack of scalability.

Given the latest advances in fiber fabrication techniques and the capabilities of complicated fiber structures like multi-core fibers^27^,^28^,^29^ and photonic crystal fibers^30^, the opportunity of implementing a neural network using such optic fibers seems both feasible and directly addresses the challenges presented above. Multi-core fibers (MCF) offer, via a simple geometric design within a small volume, conductance of many light sources with the possible utilization of splitting, convergence and amplification capabilities^27^,^28^,^29^,^31^. Here, we present an in-fiber neural network, and demonstrate, by using numerical simulations, its ability to distinguish between different input patterns.

## Materials and Methods

**Figure 1:**
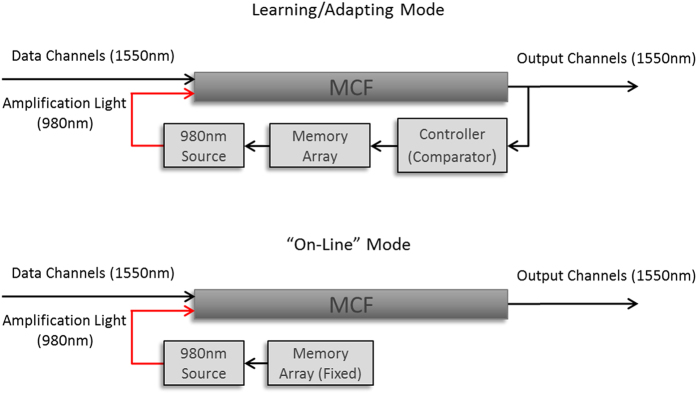
The MCF–based in-fiber neural network learns with external feedback and learning algorithm. During learning or adapting (if required), the outputs are sampled by detectors and delivered as electronic signals to a PC or a controller, which computes the necessary change in amplification pattern and replaces synaptic weights (top). After learning is optimized, the device can work “on-line” without further change of weights, thus no longer requiring feedback or a learning algorithm (bottom).

In optical neural networks light replaces the electrical signals transmitted between live neurons. The basic principle in our design was to use coupling between light-conducting silica cores as an analog for the transfer of such electrical signals. We chose to implement a feed-forward neural network with a non-trivial architecture and complexity level, while still being simple enough to enable some reasoning of the dynamics. At this stage, our feedback and learning algorithm are external to the device and include output detectors, electronic device (or a PC), which implements the learning rule, and a memory array holding weight values which control an array of amplification light emitters (Fig. 1, top). After “off-line” learning, the weights are fixed and the algorithm machinery is no longer needed (Fig. 1, bottom).

**Figure 2:**
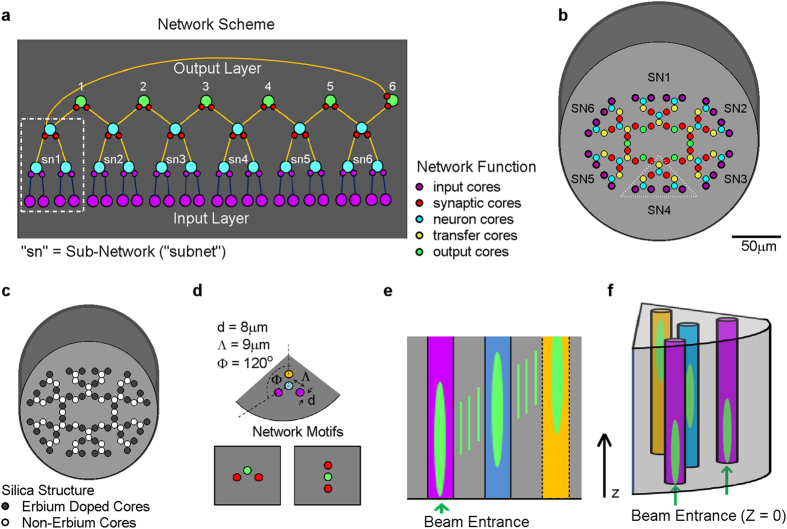
Architecture of a feed-forward network with lateral connections in the output layer. (**a**) The network may be subdivided to six subnets (SN), where the output of each is shared by two adjacent output neurons. (**b**) The multi-core fiber implementation; 90 silica cores are embedded in cladding, each core has a functional role and input cores serve also as synaptic cores. (**c**) Amplification scheme: 48 of the cores are erbium doped (dark gray) and serve as controlled amplifiers. (**d**) The microstructure of network motifs: the three types of building blocks that are used in the network. (**e**) Two-dimensional scheme depicts the tangential signal transduction; coupling between neighboring cores simulates the transfer of signals in the network while propagation along z-axis simulates the passage of time. (**f**) Three-dimensional illustration demonstrates simultaneous coupling and convergence of two cores to one.

Our network structure (Fig. 2a) includes 24 neurons at the input layer (magenta), 12 neurons at the first layer (light blue), and 6 neurons at the second layer (light blue). The structure of the input, first and second layers, is naturally divided into 6 functional subnets (SN), each receiving 4 inputs. The output layer also consists of 6 neurons (green) each receiving inputs from two subnets, sharing them in a convolutional manner with its neighboring output neurons. So from the point of view of the outputs, each reflects a function of 8 input channels.

In order to maximize volume usage, we chose a converging network where the input cores are set on the outer perimeter of the MCF and outputs are the most central. Note that the outermost input cores function also as synaptic cores (Fig. 2b). Therefore, while our network would eventually be implemented as a 3D device, the actual functionality of the network lies only in the xy-plane, and the propagation of light along the z-axis represents the time course of activity propagation through the network.

A central characteristic of artificial neural networks is their ability to shape the connection mapping (gain) between existing nodes. Specific synaptic weights, which set the gain of each input to a real biological neuron, are mimicked in our model by Erbium-doped amplification in cores that interconnect input cores with neuron cores (synaptic cores, Fig. 2c top). Core diameter was set to d = 8 μm in order to optimize mode area in the device and the pitch was set to Λ = 9 μm to enable proper coupling of the signal light at 1550 nm, while minimizing the coupling of pump light, 980 nm (see more details below). Using refraction indices of commonly found commercial multi-mode fibers (core index = 1.52, cladding index = 1.48), we obtained fibers with a theoretical NA = 0.35, and a critical angle ϴ_c_ = 20.27°.

The network is built as a combination of three connection motifs: “2 to 1 to 1” (Fig. 2d, top), and two types of “1 to 1 to 1”; in the former the angles are 120 degrees (Fig. 2d, bottom left) and in the latter the 3 cores are aligned (Fig. 2d, bottom right).

Light sources (with Gaussian power in xy-plane) were set at one edge of the device (z = 0) and light beams were set parallel to z-axis (Fig. 2e). As beams propagate along the z-axis they also couple tangentially in the xy-plane into neighboring cores. Thus, due to the converging design, electro-magnetic energy is shifted centripetally toward more internal cores while propagating along the z-axis (Fig. 2f). After several such inward couplings and convergences, the light beams are coupled with the inner most neuron cores, which serve as outputs of the device (Fig. 2b marked green). The length of the fiber was set to be 9 mm, because at this length, light reaches the output cores, and does not couple in the opposite direction. This length, however, is a function of the coupling distance between cores, and may be set differently if more complex dynamics are intended (e.g. addition of recurrent signal flow).

Our design is based on a simple multi-core structure with identical sized cores and pitches. In order to gain an insight into the expected light propagation in the device we considered the standard coupled-mode equations^32^,^33^. We describe here the equations that are relevant for the three motifs (Fig. 2d). It is of interest to note, however, that while these equations are useful for understanding the process, they were not used to generate the results; rather the full 3D wave propagation equations were computed (see below).

The three motifs are (as described in Fig. 2d):

1. Three cores surrounding a central core with 120° between them (top).
2. Same motif as in (1) but with one of the surrounding cores omitted (bottom left).
3. Straight motif of three aligned cores (bottom right).

For the first motif the equations take the form of:


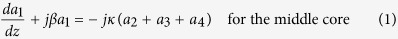



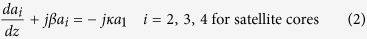


where *κ* is the coupling coefficient (equal to all cores), *a*_*i*_ is the fundamental mode of core *i*, and *β* is the propagation constant of the fundamental mode in the cores. Similarly for the second motif (two cores in 120 degrees):


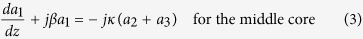



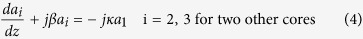


and for the last motif, with the aligned cores we assume identical equations as in the second motif but with a slightly different coupling coefficient *κ*_*a*_ ≈ *κ* due to the difference in angle, and thus slightly longer distance between external cores (d_1,3′_ = 2* Λ instead of d_1,3_ = 2* Λ*cos(30°); d_1,3′_/ d_1,3_ ≈ 115%, but as d_1,3_ ≈ 2*Λ, we assume negligible crosstalk between the two external cores).

We assume the following boundary conditions for the first motif:


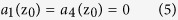


Assumption: entries of propagating signals are via cores 2, 3 first. For input cores z_0_ = 0 but for other cases z_0_ may be larger. Direction of energy flow through the cores:

2 = >1 = >3 and 4, and 3 = >1 = >2 and 4.

Similarly, for the other two motifs:


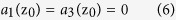


Assumption: entries of propagating signals are via core 2 first. Direction of energy flow 2 = >1 = >3. As polarization independence is assumed, the solution is of the form:


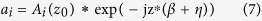


Introducing [Eq. 7] into [Eqs 1, 2, 3, 4, 5, 6] permits the eigenvalues *η* to be obtained.

Implementing a neural network implies that the power at each neuron core follows this general rule:


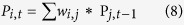


where *P*_*i,t*_ is the power of neuron *i* at time *t*, 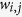 is the synaptic weight (or gain) of inputs from neuron *j* to neuron *i*, and *P*_*j,t*−1_ is the output of neuron *j*, generated at time *t − 1*. A learning network implies that the set of 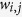 are modified and refined in an iterative process until the desired input-output mapping is achieved.

As we mentioned earlier the full analysis of our MCF configuration requires a numeric solution of the 3D wave propagation equations. We have used RSoft simulation software (Synopsis, California, USA) in order to carry out these computations for all the simulations presented in this study. The actual equations solved in the simulations are the wave equations with boundary conditions that include all the details and actual sizes of the structure (e.g. pitch of 9 μm and specific angles). We have used RSoft software which implements a finite-difference beam propagation method^34^,^35^,^36^. To address non-linear effects we have carried out several simulations including non-linearity and dispersion effects. However, no major differences were found (results not shown). This is mainly because of the short length of the device, low power input signals and a CW source^37^. Therefore, in the simulations we assumed we could neglect non-linear effects and, indeed, our experimental results confirmed this. For modal analysis we used RSoft tools which implement two types of algorithms: correlation method and imaginary distance method. The correlation method uses the Fourier transform of the correlation between input field and propagating field to compute the coefficients of each mode^34^. The imaginary distance method is an iterative algorithm in which propagation in z-axis is substituted with loss/gain (z′ = iz). In each iteration a “winner takes all” mode is computed and subtracted by orthogonalization before the next iteration^38^.

## Results and Discussion

**Figure 3:**
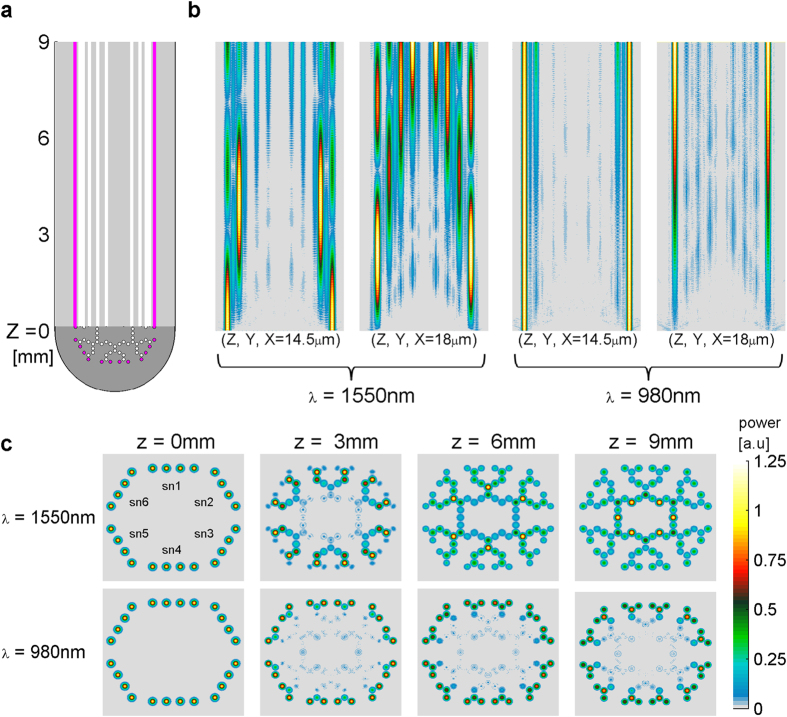
Light coupling between cores is different for 1550 nm and 980 nm. (**a**) yz-plane cross section of the MCF at x = 0: magenta circles and lines denote input cores. (**b**) Signal propagation along yz-planes at (z, y, x = 14.5 μm) and (z, y, x = 18 μm): coupling of light from outer cores to more central ones is more effective in 1550 nm than 980 nm. (**c**) Cross-sections along the z-axis clearly demonstrate the centripetal coupling and the differences between the two wavelengths. Notice the absence of trans-synaptic contamination; amplification of adjacent synaptic cores does not mix.

Light sources (Gaussian shaped power in the xy-plane) at wavelengths of 1550 nm (signal) or 980 nm (pump) were applied at the input cores (z = 0) in the simulation (Fig. 3a). Light propagated along the z-axis and also spread in the xy-plane due to coupling between adjacent cores. One main goal in the design was to differentiate the coupling of signal light and the coupling of pump light. While the signal light intended to couple from one core to its adjacent cores, the ideal pump light should remain confined to its injected core. Here, we aimed to minimize the coupling of 980 nm light and, as much as possible, to avoid cross-amplification from one “synapse” core to others. Simulation results in the yz-plane shows that signal light (1550 nm) propagated by coupling across seven adjacent cores along the fiber length, while the pump light effectively propagated only to the neighboring cores (x = 18 μm or 14.5 μm denote the yz-plane Fig. 3b). As expected, the results demonstrate the pronounced difference in inter-core coupling between the two wavelengths (Figs 2c and 3b).

Pump light power declined linearly along the z-axis to about 70% of its initial power (Fig. 3b). However, in the 1550 nm simulation, we set the amplification to linearly decline to 50% of its initial value at z = 9 mm, as an underestimate, considering possible additional losses. We also set the pump light in the most internal ring of cores applied in the opposite direction; beams are set at z = 9 mm and propagate toward z = 0. This was done to increase the efficiency of output amplification. For demonstration purposes only all amplifications were set to a high value of 850 dB/m (elaborated in the discussion).

**Figure 4:**
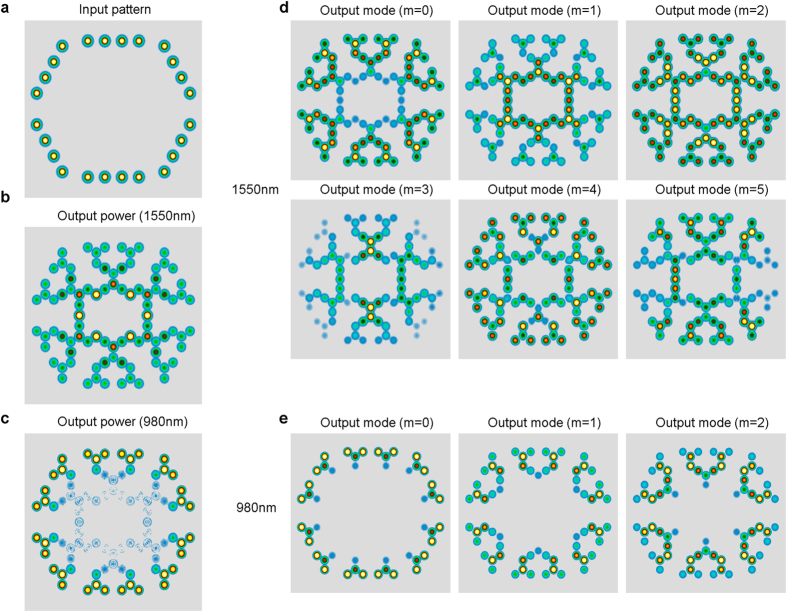
Modal analysis exhibits higher modes propagation. Simulation was done with the simple symmetric input (**a**) and its corresponding overall output at 1550 nm (**b**) or 980 nm (**c**). (**d**) Modal analysis of the 1550 nm demonstrates 5 propagating modes, where the fundamental mode (m = 0) does not propagate well beyond layer 2 in the network whereas modes (m = 1, 2) propagate all the way to output layer. (**e**) In contrast, 980 nm modes are confined to layer 1–2, similar to the overall output power.

Due to the fact that we used indices that enable multi-mode propagation, we conducted full modal analysis using the RSoft tool (see materials and methods for details). The modal analysis demonstrated that symmetric input beams (Fig. 4a), which reach the output core at z = 9 mm (Fig. 4b) are propagating as the combination of several modes (Fig. 4c) with different properties. While the fundamental mode is mostly restricted to layer1 and 2 of the network, modes m = 1 and m = 2 reach the output layer and contribute to the output power. Higher modes show asymmetry in the xy-plane. Replacing the 1550 nm by 980 nm inputs (output shown in Fig. 4d) shows fewer modes that are all confined to the first two layers and shows a very small possibility for cross-synaptic amplification (Fig. 4e).

**Figure 5:**
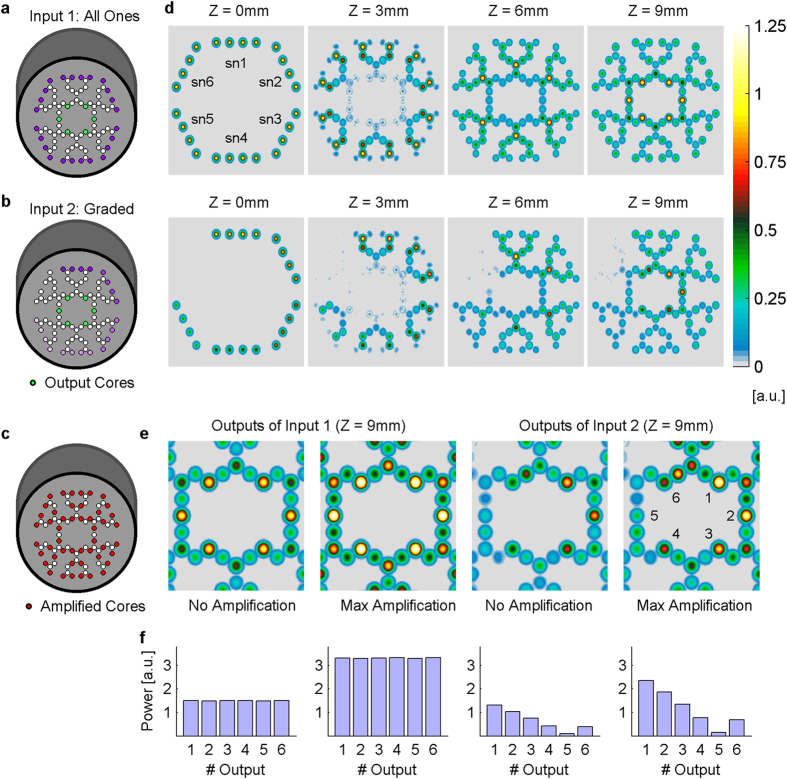
Transmission in the network for various input patterns. Two input patterns are compared: (**a**) “all 1” where all inputs are maximal, and (**b**) “graded” where the inputs of each subnet have a fixed power of [100% − (n − 1) * 20%], where n is the subnet number. (**c**) Simulation results of the two different input patterns at various z values with no amplification shows the circular symmetry in the “all 1” inputs, and the power gradient preservation along the z-axis in the graded input. (**d**) Illustration of all amplified synaptic-like cores. (**e**) Addition of amplification results in similar output ratio with additional “DC” power. (**f**) Quantified results of each output core clearly demonstrate that amplification does not affect the ratios between outputs in these two examples. Notice that core 6 has lower power than expected due to its averaged inputs (50% as the inputs of core 3).

To test the propagation of an asymmetric input, we next compared an input pattern of maximal inputs (“all 1”, Fig. 5a) with graded input (0, 20%, 40% … 100%, Fig. 5b). Simulation of “all 1” input pattern demonstrated concentric symmetry in propagation along the z-axis (Fig. 5d, top row) and in the output plane as well (z = 9 mm). In the graded input simulations, output power was proportional to the average of eight input powers affecting each output (Fig. 5d, bottom row). So far these results exemplified the input-output relationship when all synaptic-like amplifications are set to zero. When amplification was set to maximal value (locations are marked in Fig. 5c), similar outputs ratio resulted, with addition of proportional output power (Fig. 5e). An exception to the correlation between average input power and output power, where graded input is concerned, was output 6 (it receives inputs from sn6 and sn1); with 4 × 0% and 4 × 100%, respectively. While the output power was expected to correspond to the average input power of 50%, similar to output 3, it was actually more similar to output with an average input of 30%, like output 4 (Fig. 5e right). Such seemingly non-linearity is further discussed below. The quantified relative power of each output core in each condition is presented in Fig. 5f.

**Figure 6:**
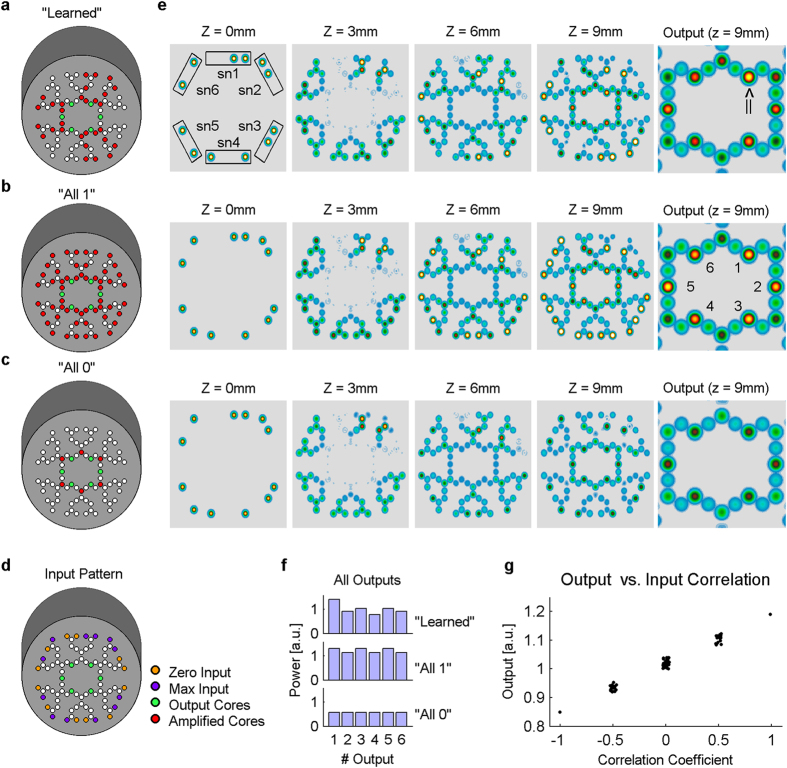
A network that classifies input patterns. (**a**) “Learned” is a specific amplification map which mimics a device after training for a specific searched input vector ‘00111100’ and is replicated 3 times within the network. (**b**) A control “all 1” homogeneous amplification map contains no embedded information. (**c**) A homogeneous control “all 0” amplification map also contains no embedded information. (**d**) Three 8-bit input vectors are tested simultaneously: the searched pattern is tested at output 1 (presented to sn1+sn2 subnets), and two control vectors are tested at output 3 (sn3+sn4), and output 5 (sn5+sn6). (**e**) Simulation results of 3 vectors with 3 amplification maps shows that only the “learned” amplification map distinguishes the searched vector from the rest. (**f**) Quantified output power of the three simulations demonstrates the difference between output 1 and outputs 3 and 5. (**g**) Simulation of all possible 8-bit input vectors with 4 “zeros” and 4 “ones” with the “learned” configuration: the output power grows larger as its 8-bit input is more similar to the “searched” pattern (higher coefficient of correlation).

A hallmark of artificial neural networks is pattern recognition, classification, and learning. When a neural network learns an association between a set of inputs and the desired outputs, the parameters that are being changed are the synaptic weights of each connection in the network. In a multi-layer feed-forward network, such as considered here, there are many algorithms to achieve this goal. Most notably is the algorithm known as back-propagation^39^,^40^, which updates the synaptic weights of each layer according to a computed error. The algorithm starts at the output layer, computing the error by comparing the actual output to the desired one. This error is then propagated back to preceding layers, modifying the weights at each layer accordingly. Note that once a desired minimal error is achieved, no further changes are made to the synaptic weights and the input-output relationship is fixed and could be used for pattern classification. In the context of this work we chose not to simulate the learning process itself as it is beyond the scope of this research. Rather we show that choosing a specific non-trivial set of weights (in our case specific values in the amplification cores), a non-trivial input-output mapping is obtained (see below Fig. 6). This suggests that by changing the values of the amplification cores with an iterative process similar to back-propagation, our device could implement a learning algorithm. We tested whether the network can identify a specific input pattern, as if it had been previously trained for this task. The goal was to identify the arbitrary pattern “00111100” (denoted further as the “searched” pattern) out of other possible patterns with similar statistics (4 ‘zeroes’ and 4 ‘ones’). The synaptic-like configuration that was guessed as appropriate for the identification (i.e. “learned”) was suited to the “searched” input pattern, with maximal amplification in specific connections and no amplification in all others (Fig. 6a). This configuration was duplicated in a symmetric fashion in respect to output core 1, output core 3 and output core 5 (for input patterns presented to sn1+sn2, sn3+sn4, and sn5+sn6 respectively). We compared this configuration of amplification to two other control configurations: “all 1” – namely, all signals through “synaptic cores” are maximally amplified (Fig. 6b), or “all 0” - meaning none is amplified (Fig. 6c). In the given example, the “searched” pattern was presented to sn1+sn2, while sn3+sn4 and sn5+sn6 were presented with two other 8-bit input patterns (Fig. 6d). Thus, the corresponding outputs for comparison were output core 1 (for the “searched” pattern input) versus output cores 3 and 5. This input configuration was simulated with each of the above mentioned amplification configurations.

The simulation demonstrates that when the connectivity is the “learned” configuration the output power differentiates the “searched pattern” from the other input patterns (compare output core 1 to output core 3 and 5; Fig. 6e, top row right, denoted by arrow). However, no such specificity shows up in the control configurations (Fig. 6e). Quantification of the results supports the graphical result (Fig. 6f).

To examine the generality of this result we ran the simulation for all possible input combinations with 4 ‘zeroes’ and 4 ‘ones’. Figure 6g depicts for each such input pattern the amplitude of the output unit against the correlation coefficient between the input pattern and the “searched” pattern (00111100). As shown, there is a linear relationship between these two quantities suggesting that the output power indeed reflects the extent that the input pattern resembles the “searched” pattern, and the potential of the network to classify input patterns.

**Figure 7:**
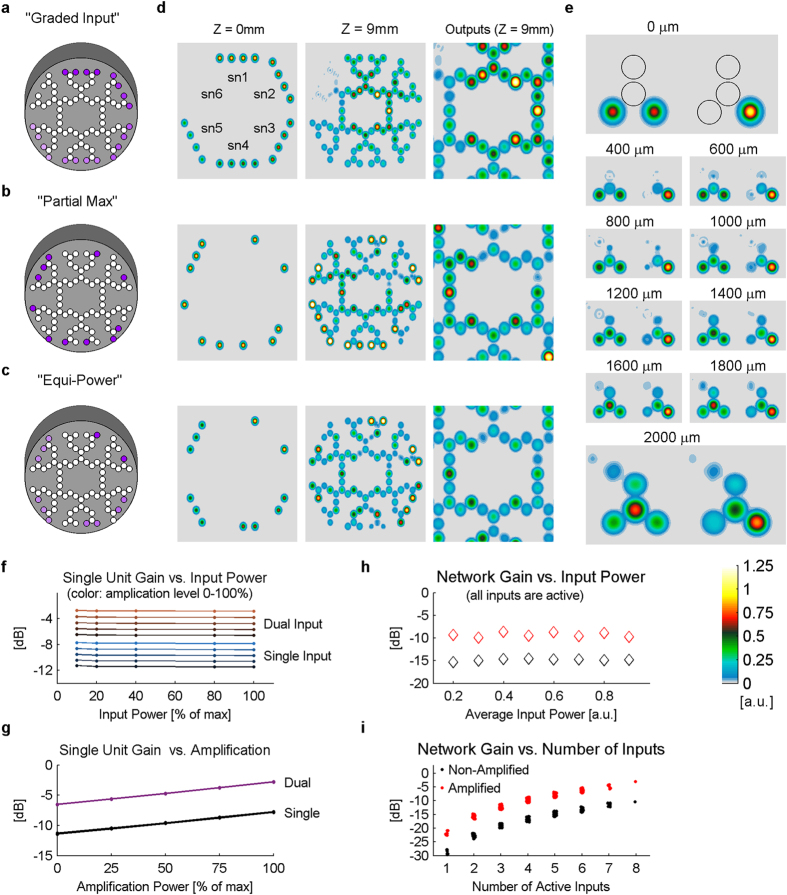
Synergistic power transmission for different input combinations. Different input patterns with partial inactive channels were tested: “Graded” input (**a**) was compared with “Partial Max” (**b**), where all non-missing inputs are at maximal power and “Equi-power” (**c**), where the sum of all 8 inputs to outputs 1, 3 and 5 is kept constant at 1 and the number of active inputs increases. Simulation of all three input patterns showed deviant coupling with a high portion of power that did not progress toward the center of the device but rather remained at the input cores (**d**, center column). Simulation of a single unit when either two sources (of 50% power) or a single source (100%) are given as inputs (**e**) demonstrates the difference in light propagation between the two configurations. Deeper analysis of the input-output relationship of single units (**f**,**g**) shows that output gain is linearly dependent on amplification level and on symmetry of inputs (as a constant gain increase for 100% vs 0% symmetry). At the network level: when no input is omitted (100% symmetry), overall input/output ratio is almost constant and depends on synaptic amplification (**h**), while simulations results of all input space shows a sharp drop in output gain as more inputs are omitted (**i**).

We have previously shown that output core 6 demonstrated a “sub-linear” result when 4 of its inputs were set with maximal amplitude and the others were set at zero (Fig. 5). In order to examine this sub-linearity in output gain, we compared two new configurations of inputs to the graded one (Fig. 7a): one where the number of non-zero inputs is different for every output and all existing inputs have maximal power (“Partial Max”, Fig. 7b), and one where the number of non-zero inputs is changing while keeping the average power constant (“Equi-Power”, Fig. 7c). The simulation results (Fig. 7d) showed that indeed, when several inputs are omitted, the flow of energy via coupling between the cores is prevented, and instead of propagating forward to the next layers of the network (more central, e.g., Fig. 7d top), much of the energy remains in the input layer cores (Fig. 7d middle and bottom). Detailed examination of the equi-power simulation (Fig. 7b bottom) reveals that light propagation towards the output cores is proportional to the number of inputs rather than the level of the input power. The results of the simulation suggested that coupling of light towards the output cores is assisted by a high level constructive interference between phase-locked input sources, indeed simulations with phase shifted sources showed reduced output gain (not shown here). We conducted a close examination of energy propagation through a single unit comparing a unit with a single 100% input vs. a unit with two 50% inputs, sampling the propagation every 200 μm (Fig. 7e). The results indicate that propagation of light up to 800 μm (in z-axis) shows no large difference between the two configurations. However, at 1200 μm and beyond, the results split: most of the power in the unit with the symmetric inputs is transferred from the input core to the next one, while in the asymmetric unit, most of the power remains in the input core where it started. Further examination of this difference, by simulating the outputs of single units, showed that the gain of a single unit is independent of input magnitude, but depends linearly on amplification level (Fig. 7f,g). The results also showed that symmetric inputs have output gain larger by 4.92 ± 0.06 dB as compared with single input of the same magnitude, regardless of amplification level (Fig. 7g). At the network level, quantification of the graded input simulation (Fig. 7h) demonstrated a constant amplification when all inputs are activated, and share similar power. In contrast, quantification of all possible combination of partial inputs (with binary values of zero or max) shows clear sub-linear gain when some (or many) of the 8 inputs to a single output are omitted (Fig. 7i).

**Figure 8:**
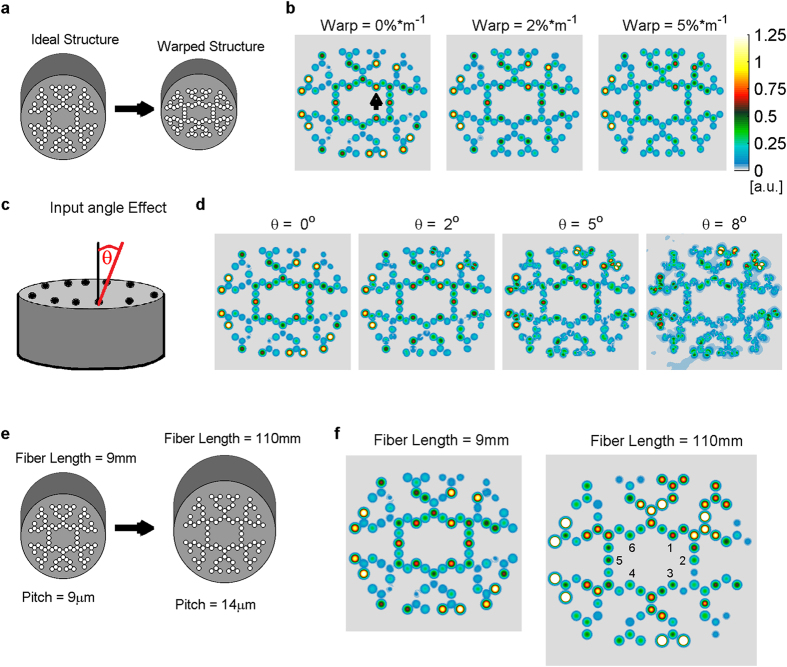
Pragmatic stability of network function. Practical considerations such as fabrication defects, input angle deviation and lower amplification level may affect the function of the network. In all three examinations we compared the output of the altered device with the ideal one, using the results of pattern identification (as shown in Fig. 2). (a) Generating a stress-like warping at random angle (in the xy-plane) with random global changes in core and cladding indices did not have a major effect on the relative output reading (output 1 vs. 3 vs.5 see arrow marking) even when deformation was taken beyond what is assumed as a standard fabrication capability (1–2% deformation per meter). (**b**) While all previous simulation assumed that input sources re all parallel to Z-axis (ϴ = 0°), we show that mild tilting of sources (up to ϴ = 8°) still enable adequate relative outputs (all weaker but ratios are maintained). (**c**) We previously assumed that given weaker and more realistic amplification power, we can design a longer device with larger pitch that will retain the same function. Here we demonstrate such a device that is 12 times longer, with 20 times weaker amplification, and 14 μm pitch instead of 9 μm. Again, while overall output is weaker, the ratio between output cores is maintained (output 1 ≫ output 3 or output 5).

The last group of simulations was dedicated to examine how pragmatic considerations may affect the functional robustness of fabricated networks. We firstly examined fabrication uncertainty. As we could not cover all parameters that might affect the device function due to fabrication, we focused on several quasi-realistic parameters that we could introduce in the simulation. Some parameters that potentially could be compensated for by tuning the light intensities for individual cores (for example amplification parameters such as Erbium ion concentration) were not included in the analysis. We manipulated the design such that a quasi-realistic deformation occurs along a random axis in the xy-plane, as if a constant uneven pressure was applied during the pulling of the fiber (Fig. 8a). This deformation changes the location of each core in proportion to its distance from the main deformation axis. Additionally, the overall length of the device, and the indices of both core and cladding were also subjected to random noise (up to 5%) within the same design. We used the pattern identification results (Fig. 6) as a benchmark for the network’s functionality. The results of these simulations showed that even when parameters were subjected to a variance of 5% per meter (normal fabrication deformation is ~2–3%/m or better), the results were still robust. Although the overall output power is decreased, the ratio between outputs 1, 3 and 5 remained similar (Fig. 8b, the output cores are numbered below in Fig. 8e). Examining the effect of input light tilting, we showed that up to an angle of 8° network outputs are still robust, maintaining output ratios while overall power is decreased (Fig. 8d). Lastly, we designed a longer device with amplification that is 20 times weaker (~41 dB/m, Fig. 8e). Results show that if the pitch is set to 14 μm (Fig. 8), the coupling distance is increased in proportion to the length such that the spatial pattern of relative power at the output cores is qualitatively maintained (Fig. 8f).

**Figure 9:**
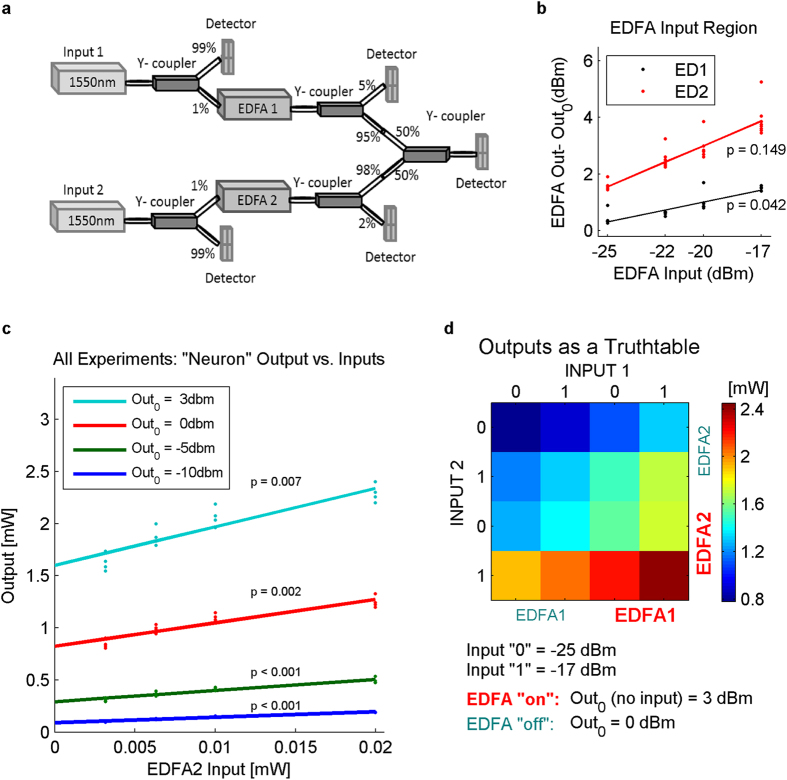
Experimental validation of our conceptual “neuron-like” unit. (**a**) We implemented one in-fiber neuron unit which comprises two separately amplified input cores and an integration core using single mode fibers, Y-couplers and two EDFAs. (**b**) We chose low power inputs so that the amplifiers will function within their linear amplification region. EDFA outputs without inputs (Out_0_) were subtracted from all outputs with inputs to examine the effect of inputs (ED = EDFA). The y-axis variance stems from the different levels of amplification. (**c**) The “device” output shows linear dependency on EDFA2 inputs while EDFA1 inputs contributed mostly to the y-axis variance. It is clear that while stronger amplification results in greater influence of the inputs (cyan graph), linearity in weak amplification becomes sub-linear with stronger amplification. (**d**) Combining two levels of inputs and two levels of amplification into a color-mapped truth table demonstrates that both input level and amplification level determine the output of the modeled unit.

The basic characteristic of any artificial neuron is that its output is a function of the weighted sum of its input (each multiplied by a specific gain factor). Our simulation results show that combining inter-core coupling with specific amplifications level can generate such a computational network. To test if this basic principal holds in real hardware we constructed our basic building block unit with two CW lasers (1550 nm), two Erbium-doped fiber amplifiers (EDFAs) and five y-couplers. Single-mode fiber connectors were used for connections and several power detectors to monitor power in different stages of the circuit (Fig. 9a). The amplified input cores of each unit in our network were implemented by using a laser source and an EDFA; the integrating core (“soma”) was modeled by 50–50% 2:1 y-coupler (right most coupler in Fig. 9a). All other couplers were used for signal readout and calibrations.

As EDFA amplification is quasi-linear for small signals and sub-linear (saturating) for strong signals, we chose our inputs to be weak. Therefore, the input to each EDFA was taken at the 1% output of the source readout coupler (Fig. 9a) and the set of inputs were chosen to get maximal linearity (−25, −22, −20 and −17 dBm, Fig. 9b). Notice that quasi-linearity is seen after removing the base noise level (Out_0_, output when no input is introduced). The EDFAs were operated at 4 levels according to their Out_0_: Out_0_ = −10, −5, 0 or 3 dBm. 256 combinations of all possible inputs and amplifications levels were measured at the output of the integrating coupler.

The results for equal level of Out_0_ for the two EDFAs are shown in Fig. 9c; the effect of input_1_ variation is presented as several data points at each node of Input_2_. For example: for input_2_ = −20 dBm (0.01 mW), and both amplifiers are at Out_0_ = 3 dBm (cyan) the bottom dot represents input_1_ = −25 dBm while the top one represents input_1_ = −17 dBm). It is clear from the graph that input_2_ had a stronger effect on the output than input_1_, due to the differences between the amplifiers.

To examine the interaction between input powers and amplification levels we minimized the input and amplification space to a binary option for each parameter. In the truth table for all 16 options (Fig. 9d) the color-coded results immediately show that both input level and amplification level of each input contribute to the variance of the final output.

**Figure 10:**
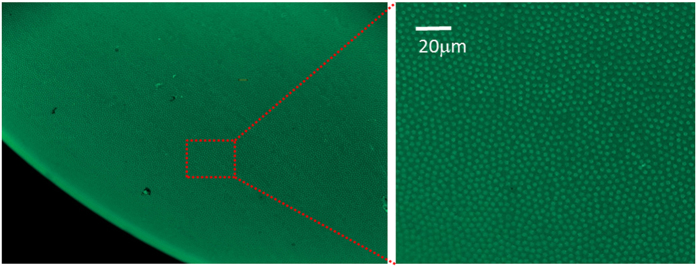
MCF Fabrication capabilities. In parallel to our design, simulation and testing basic hardware configurations, our group also developed the capability to fabricate a polymeric MCF with ~750,000 cores. The cores have a diameter of are 0.9 μm and a pitch of 3.6 μm, with a core index of 1.59 and a cladding index of 1.49. Future experiments will include testing our designs with such polymeric MCF.

To further support the feasibility of our device our group, in collaborative efforts, developed fabrication capabilities and succeeded to construct a polymer based MCF (Fig. 10). The polymer consists of the indices: n_core_ = 1.59 and n_cladding_ = 1.49. This MCF may contain as many as 750,000 cores, each with a diameter 0.9 μm and pitch of 3.6  μm. Future research will focus on a neural network design and implementation in such polymer MCFs devices based on the principles laid out in the present study.

In this study we have shown that our network provides the means to carry out useful classification and learning. In this context it is useful to distinguish between the two. To demonstrate classification, we need to show that the network provides different outputs for different input patterns. In its minimalist form, provided that we can “tag” any input pattern with a binary tag (i.e. 0 or 1), we impose a classification on the input patterns. However, such classification could be completely random and meaningless and therefore, on its own, is not very interesting. Thus, in Fig. 6 we show that our network can tag input patterns with a meaningful tag. We designed this specific network such that the output is a similarity measure between the input pattern and a specific target pattern. We measure the similarity between the input patterns using a bit-to-bit correlation with the target pattern. We show that input patterns that are similar to the target pattern provide a strong output when introduced to the network and input patterns that are dissimilar have a weak output. We show that for the set of all possible input patterns that have the same number of “on” bits in the pattern (total of 4). This demonstrates that our network is capable of doing a meaningful classification computation.

The essence of learning in such a network means that we should be able to change the synaptic weights until we reach the set of weights that achieves the desired classification. So the two questions at hand are: (1) Can we change the weights in the network over a wide dynamic range? (2) Do we provide a learning algorithm that can converge to the desired set of weights? The answer to the first question is yes. The synaptic weights in our model are controlled by the amplification of the dedicated cores. As we show in the simulations (Fig. 3), and now using the experiments in Fig. 9, changes to the power of these cores dramatically changes the behavior of the network and thus exploring different patterns of 980 nm light that fed into the “synaptic cores” is equivalent to a search through the hyper-dimensional space of synaptic weights in an artificial neural network. As for a learning algorithm (question 2), we are not committed at this stage to a specific learning algorithm for our network. Instead we provide a flexible system and the means to implement any of a variety of possible algorithms that can be tested, and we demonstrate in a simple example that at least for one case a solution exists. In this context, it is important to note that artificial neural networks do not promise convergence on all possible cases (e.g. the perceptron learning rule and the XOR problem^41^). In some cases, network configurations have to be explored in order to achieve a good classification, and as for the current research status this is more an art than a science. Lastly, it is worth noting that using optical networks provides a significant advantage because the increased speed of such networks can even make the use of an inefficient learning algorithm useful.

## Conclusions

We present a novel conceptual framework for implementation of an in-fiber multi-layer neural network within a modest size MCF. Our simulation results demonstrate that in such a network 980 nm pump light is confined to one or two cores while in contrast 1550 nm signal light travels to other cores due to better coupling. The output units of the network integrate and amplify input power, and we demonstrate that computations such as input classification might be implemented based on this property.

The network also shows a complex interaction between the spatial distribution of power over the input units and the final output power distribution. We note that while in this study we treat it as a setback, it may actually be utilized in the future as a feature, for example to implement sigmoidal functions, or even inhibitory dynamics. We have partially explored the susceptibility of the design to fabrication uncertainty. We found that our specific design is resilient, and may function well even under strong misshaping, inaccurate light source angle or, if elongated, to allow for more realistic amplification. Moreover, we carried out experiments with physical devices and show that given an input space of weak signals, quasi-linearity may be achieved for each amplified input, and the integrated output is sensitive both to amplification level and to each input level, supporting our previous assumptions. Lastly, we demonstrate our ability to manufacture a high density polymer MCF that may be utilized to implement such devices in future.

MCFs can be used as basic architecture for designing numerous general or computation-specific neural networks. While many obstacles may present themselves in the subsequent prototypical experiments, we show here that this type of device is conceptually valid and producible. The presented network connectivity structure is designed to be a simple configuration only for demonstration purposes. Larger MCF with up to ~10^6^ cores can house not only many more neurons and synapses, but also more complex neurons each with dozens or hundreds of synapses which connect with non-trivial convergent and divergent structures. It is likely that combining the centripetal propagation of power used in this design with a centrifugal propagation will allow the achievement of reverberation of activity and more complex computations. The remaining challenges that we expect are the embedding of new amplification techniques (i.e. quantum dot lasers), and the construction of electronic control, which will implement the learning algorithm via the amplification pattern. Using a realistic level of amplification in the core (i.e. 1–2 dB/m), we can still achieve a functional readout by making our device longer (10–30 m). As we demonstrated, this will require an increase in the pitch between the optical cores in order to keep the ratio of coupling distance and device length fixed. To overcome the complexity associated with modifying synaptic weights, another possible design might be to use a design based on a “fixed-weight” neural network^42^,^43^. One of the major challenges we will face is coupling of multiple external light sources into the relevant cores of the MCF. One way to approach it might be to use a fast micro-mirror array (DMD) to control the wavefront or a spatial light modulator (SLM) together with an array of micro-lenslets. For a relatively small number of fibers we can use the cone-shaped un-pulled edge of the MCF as described earlier in the literature^44^, but such a connection may become burdening for large number of inputs (i.e. >100x). As coupling to MCF is a common challenge for researchers and commercial developers, we also expect new “off-the-shelf” solutions for this problem in the future as recently presented by Furukawa Electric^45^ (Japan). Mode control presents another challenge. The importance of the specific mode excited comes from its control of the amount of coupling between the cores, however it is difficult to excite only a specific mode. In this study, we assumed that the Gaussian beam that was launched into the fiber not only generated the fundamental mode but also several higher modes as Fig. 3 suggests. In the future, if necessary, mode control could be assisted by specific phase control using a fast SLM with dedicated pixels for each of the input signals. As these challenges will be met by new research and designs, practical and useful optical computation devices are closing in on their electronic counterparts.

## Additional Information

**How to cite this article**: Cohen, E. *et al*. Neural networks within multi-core optic fibers. *Sci. Rep.* **6**, 29080; doi: 10.1038/srep29080 (2016).
